# Notch3 inhibits cell proliferation and tumorigenesis and predicts better prognosis in breast cancer through transactivating PTEN

**DOI:** 10.1038/s41419-021-03735-3

**Published:** 2021-05-18

**Authors:** Yong-Qu Zhang, Yuan-Ke Liang, Yang Wu, Min Chen, Wei-Ling Chen, Rong-Hui Li, Yun-Zhu Zeng, Wen-He Huang, Jun-Dong Wu, De Zeng, Wen-Liang Gao, Chun-Fa Chen, Hao-Yu Lin, Rui-Qin Yang, Jiang-Wen Zhu, Wan-Ling Liu, Jing-Wen Bai, Min Wei, Xiao-Long Wei, Guo-Jun Zhang

**Affiliations:** 1 Department of Breast-Thyroid-Surgery and Cancer Center, Xiang’an Hospital of Xiamen University, 2000 East Xiang’an Road, Xiamen, China; 2 Department of Breast Center, Cancer Hospital of Shantou University Medical College, No. 7 Raoping Road, Shantou, China; 3 Department of Thyroid and Breast Surgery, Clinical Research Center, The First Affiliated Hospital of Shantou University Medical College, 57 Changping Road, Shantou, China; 4 Klinikum rechts der Isar der Technischen Universität München Institut für Allgemeine Pathologie und Pathologische Anatomie, Ismaninger Str. 22, 81675 München, Germany; 5 Clinical Central Research Core, Xiang’an Hospital of Xiamen University, School of Medicine, Xiamen, China; 6 Key Laboratory for Endocrine-Related Cancer Precision Medicine of Xiamen, Xiang’an Hospital of Xiamen University, Xiamen, China; 7 Cancer Research Center, School of Medicine, Xiamen University, Xiamen, China; 8 Department of Medical Oncology, Xiang’an Hospital of Xiamen University, 2000 East Xiang’an Road, Xiamen, China; 9 Department of Pathology, Cancer Hospital of Shantou University Medical College, No. 7 Raoping Road, Shantou, China; 10 Department of Medical Oncology, Cancer Hospital of Shantou University Medical College, No. 7 Raoping Road, Shantou, China; 11 Guangdong Provincial Key Laboratory for Diagnosis and Treatment of Breast Cancer, Shantou, China

**Keywords:** Breast cancer, Prognostic markers

## Abstract

Notch receptors (Notch1–4) play critical roles in tumorigenesis and metastasis of malignant tumors, including breast cancer. Although abnormal Notch activation is related to various tumors, the importance of single receptors and their mechanism of activation in distinct breast cancer subtypes are still unclear. Previous studies by our group demonstrated that Notch3 may inhibit the emergence and progression of breast cancer. PTEN is a potent tumor suppressor, and its loss of function is sufficient to promote the occurrence and progression of tumors. Intriguingly, numerous studies have revealed that Notch1 is involved in the regulation of PTEN through its binding to CBF-1, a Notch transcription factor, and the PTEN promoter. In this study, we found that Notch3 and PTEN levels correlated with the luminal phenotype in breast cancer cell lines. Furthermore, we demonstrated that Notch3 transactivated PTEN by binding CSL-binding elements in the PTEN promoter and, at least in part, inhibiting the PTEN downstream AKT-mTOR pathway. Notably, Notch3 knockdown downregulated PTEN and promoted cell proliferation and tumorigenesis. In contrast, overexpression of the Notch3 intracellular domain upregulated PTEN and inhibited cell proliferation and tumorigenesis in vitro and in vivo. Moreover, inhibition or overexpression of PTEN partially reversed the promotion or inhibition of cell proliferation induced by Notch3 alterations. In general, Notch3 expression positively correlated with elevated expression of PTEN, ER, lower Ki-67 index, and incidence of involved node status and predicted better recurrence-free survival in breast cancer patients. Therefore, our findings demonstrate that Notch3 inhibits breast cancer proliferation and suppresses tumorigenesis by transactivating PTEN expression.

## Introduction

Breast cancer is the most common cancer in women all over the world^1^. China has a similar breast cancer incidence to that of Europe and America^2^. In the past decades, surgery, radiotherapy, chemotherapy, and molecular targeting therapy have been common breast cancer treatments, leading to a reduction in mortality^3^. This decrease in breast cancer mortality is mainly due to early detection and effective systemic treatments. Unfortunately, breast cancer patients in China are often diagnosed at a middle or late disease stagze^4^. Therefore, the development of new and effective prognostic markers and optimized treatment strategies are urgently needed to improve breast cancer treatment efficacy.

The aberrant proliferative state of tumor cells is one of the main characteristics of cancer cells, including breast cancer cells^5^. Altered cellular proliferation often leads to the acceleration of tumor development and progression. Most studies have demonstrated that Notch regulates cell proliferation^6^, apoptosis^7^, cell fate^8^, and stem cell survival^9^. The human Notch receptor consists of four different receptors (Notch1–4) and five ligands, including Jagged1-2, Delta-1, Delta-3, and Delta-4. Notch signaling pathway is activated by direct contact between cells through Notch receptor–ligand binding. Following activation, Notch is cleaved into extracellular and intracellular domains by γ-secretase^10^. The Notch1 intracellular domain (N1ICD) is transported to the nucleus, and combines with the transcription inhibitor CSL [CBF1/Su(H)/Lag-1], releases the co-repressor, and recruits co-activators (i.e., p300 and MAML1^11^) to induce the epithelial–mesenchymal transition (EMT) or cell proliferation, which affects tumor progression^5^. Notch family members play an essential role in breast cancer occurrence and metastasis. The different Notch proteins have distinct biological functions and effects in breast cancer. Our previous research demonstrated that Notch1 induced AKT and EMT by directly activating of the Major Vault Protein^12^. Zhou et al.^13^ reported that Notch4 upregulates slug and GAS1 to induce EMT and quiescence of triple-negative breast cancer (TNBC). In contrast, Kim et al.^14^ confirmed that Notch2 acts as a tumor suppressor in TNBC. Furthermore, Lafkas et al.^15^ revealed that Notch3 restricts the proliferation and clonal expansion of mammary stem and progenitor cells. Recent studies have also shown that Notch3 plays an anti-oncogene role in breast cancer development and inhibits tumor growth^16,17^.

Phosphatase and tensin homolog (PTEN) is the most common tumor suppressor initially identified by a common chromosome 10 deletion in several tumor types. PTEN deletion and inactivation by point mutations in its promoter or codons were subsequently found in many cancers, including breast cancer^18^. PTEN can regulate genome stability, cell survival, apoptosis, and other essential functions^19^. Even partial functional loss of PTEN can promote tumorigenesis and cancer progression^20^. Recent mechanistic studies have shown at least two different activities of PTEN: (1) lipid phosphatase activity, which inactivates the PI3K/AKT signaling pathway; (2) protein phosphatase activity^21^.

Numerous studies have explored the potential prognostic and predictive value of PTEN in cancer. However, due to its complex mechanism, the evaluation of gene mutations cannot fully reveal the extent of its loss of activity. In addition to gene mutation, different regulatory mechanisms have been reported to alter the expression and function of PTEN, including transcriptional regulation, post-translational modifications, and protein–protein interactions^22,23^. Currently, many studies have confirmed that cross-talk between the PTEN-AKT and Notch signaling pathways exists and plays an important role in different cancers^24,25^. Intriguingly, Notch1 is associated with the regulation of PTEN through recruitment of C-promoter-binding factor-1 (CBF-1), a Notch transcription factor, and binding to the PTEN promoter^26,27^. However, the mechanism of the interaction between Notch3 and PTEN has not been extensively studied in breast cancer.

The objective of this study was to determine whether Notch3 is a direct transcriptional activator of PTEN in breast cancer. Our results revealed that Notch3-mediated activation of PTEN inhibited breast cancer cell proliferation, migration/invasion, and tumorigenesis by transcriptional activation of PTEN. In addition, Notch3 and PTEN overexpression in breast cancer patients indicated a better prognosis.

## Materials and methods

### Tissue samples

250 primary breast tumor samples were obtained by Cancer Hospital of Shantou University Medical College from 2016 to 2018. All samples were identified as invasive ductal breast cancer by pathology. None of the patients received preoperative treatment.

### Immunohistochemistry

Slides were de-paraffinized in xylene, rehydrated, and heated in citrate buffer at pH 6 to expose the antigens. Subsequently, endogenous peroxidases were quenched with 3% hydrogen peroxide, and the slides were incubated with primary antibodies and followed by secondary antibodies. Finally, diaminobenzidine was added as the chromogen (MXB, China), and hematoxylin was used as a stain. All immunostained sections were evaluated in a coded manner by the principal author, who was blinded to the patients’ clinicopathologicdata. The preparation of specimens, immunohistochemical methods, immunohistochemical staining, and scoring of Notch3 were previously described^28^. Human breast cancer tissue was detected as positive control of Notch3. The expressions were semi-quantitatively determined according to the percentage of positive cancer cells. Staining intensity was classified as four grades: none (0), weak (1), moderate (2), and strong (3). The percentage of positive cancer cells was classified as 4 grades: 0 (0%), 1 (1–10%), 2 (11–49%), and 3 (50–100%). The total score was a product of two scores and the final score of one sample was the mean of 10 microscopic fields. The median score was determined, according to which cancers were categorized into “−” (score 0), “+” (score 1–2), “++” (score 3–4), “+++” (score 5–6). The patients with “−” and “+” were defined as Notch3 Negative-expression, and those with “++” and “+++” were categorized into Notch3 Positive-expression for statistical analysis. Cells with less than 10% PTEN staining were considered negative, and those with more than 10% brown staining were considered positive^29^.

Estrogen receptor (ER) or progesterone receptor (PR) nuclear staining of ≥1% was deemed positive^30^. Human epidermal growth factor receptor 2 (HER2) determination was based on the recommendations of the HER2 testing Expert Panel^31^. We defined the cut-off value for Ki-67 as 20%. The different breast cancer subtypes of this cohort were defined as follows: TNBC: ER−, PR−, HER2−; HER-2-enriched: ER−, PR−, HER2+; Luminal A: ER+ and/or PR+, HER2−, low Ki-67 expression; Luminal B: ER+ and/or PR+, HER2−, high Ki-67 expression. The antibodies used for immunohistochemistry are listed in Supplementary Table S1.

### Cell culture, transfection, and reagents

All cell lines were purchased from the American Type Tissue Collection (ATCC) and cultured according to the manufacturer’s instructions. The cell lines were certified and negative for mycoplasma. For Notch3 or PTEN knockdown, specific siRNAs targeting Notch3 or PTEN and control siRNAs were purchased from GenePharma (Suzhou, China). The siRNA sequences are listed in Supplementary Table S2. pCLE-N3ICD (Plasmid 26894) and pCLE (Plasmid 17703) were obtained from Addgene (Cambridge, MA, USA). The PTEN plasmid (Cat: HG10421-ACR) was purchased from Sino Biological (Beijing, China). Notch3-silenced cell lines, MCF-7-shNotch3, MCF-7-shNotch3 + PTEN overexpressing, Notch3- overexpressing cell lines, MDA-MB-231-luc-N3ICD, and MDA-MB-231-luc-N3ICD + PTEN shRNA were generated by stable transfection with a silencing vector (pGPU6/RFP/Neo-shNotch3 or pGPU6/RFP/Neo-shPTEN) containing a Notch3 or PTEN targeting sequence, and plasmid pCLE-N3ICD (Plasmid 26894) containing the activated Notch3 intracellular domain (Supplementary Table S2). For transfection experiments, transfection reagent Lipofectamine® 3000 (Thermo Fisher, USA) was used according to the manufacturer’s instructions. Transfectants were selected with 1 μg/mL G418 in the medium 2 days of transfection.

### Reverse transcription and PCR analysis

The protocols of total RNA extraction and purification, cDNA reverse transcription, and RT- PCR were described previously^32^. All primers used for the PCR analysis are shown in Supplementary Table S3.

### Immunofluorescence

Immunofluorescence in MCF-7 and T-47D cells was performed as previously described^32^. Staining was visualized with a Zeiss microscope (Zeiss, Oberkochen, Germany). Notch3 and PTEN antibodies are listed in Supplementary Table S1.

### Chromatin immunoprecipitation assays

ChIP was conducted according to our previous study^32^. The PCR-amplified products (Region 2 located from –1322 to –1142 bp; Region 3 located from –905 to –702 bp) from the PTEN promoter contained CSL-binding elements (TGGGAA)^5^. One pair of primers was designed for a region without any CSL-binding elements (Region 1 located from –1470 bp to –1292 bp) as a negative control. The antibodies and primers used for the ChIP experiment are displayed in Supplementary Tables S1 and S3.

### Transient transfection and luciferase reporter assays

The PTEN promoter region containing a CSL binding site (TGGGAA) was cloned into the NheI/BgIII sites of pGL3-basic (Panomics, Fremont, CA, USA), and designated PTEN WT. The mutant PTEN promoter (PTEN MT) construct was generated in pGL3-basic with a deleted CSL-binding site (TACTAA). The vector pRL-SV40 (Promega, Fitchburg, WI, USA) was used as control to achieve the standardization of transfection efficiency. To study the effects of silencing or overexpressing Notch3 on PTEN activity, PTEN WT or PTEN MT was co-transfected into MDA-MB-231 or MCF-7 cells with different concentrations of Notch3 siRNA or vector. The activity of luciferase was measured using the Dual-Luciferase® Reporter Assay System (Promega, USA).

### Western blotting

Western blotting method was performed as previously described^33^. The antibodies used in this study, including vendors, product numbers, and dilutions, are provided in Supplementary Table S1.

### Cell proliferation assay

MDA-MB-231-luc and MCF-7 cells were seeded at 1 × 10^3^ cells/well in 96-well microplates. Cell proliferation was detected by Cell Counting Kit-8 (Beyotime, Jiangsu, China) from 0 to 5 Days. The absorbance was measured using a microplate reader (Bio-Tek, Vermont, USA). At least three independent experiments were conducted.

### Cell migration and invasion assays

Migration and Matrigel invasion chambers (BD, Franklin Lakes, NJ, USA) were conducted for migration and invasion experiment. After starvation for 24 h, 5 × 10^4^ MCF-7 cells or 2 × 10^4^ MDA-MB-231-luc cells were seeded in the upper chamber in serum-free medium. The bottom chamber was filled with the medium containing 10% serum (200 µL). The mean cell number in five fields was analyzed to determine the number of migrating or invading cells. At least three independent experiments were conducted.

### Colony formation assay

MDA-MB-231-luc and MCF-7 cells were seeded at 600 cells/well in 6-well plates. After 2 weeks, the cells were stained with Gentian Violet and counted. Three independent experiments were performed.

### Tumor xenograft models

Animal experiments followed the guidance of the Institutional Animal Care and Use Committee of Medical College, Shantou University. Six-week-old female Nu/Nu nude mice (GemPharmatech, Jiangsu, China) were used in these experiments. MDA-MB-231-luc-NC, MDA-MB-231-luc-N3ICD, or MDA-MB-231-luc-N3ICD/shPTEN cells (2 × 10^6^) were randomly subcutaneously injected into the right thigh of nude mice. Tumors were measured (length and width) every 3 days. Tumor volumes were calculated as follows: length × width^2^ × 0.5. Changes in tumor growth were monitored using the IVIS Kinetic Imaging System (PerkinElmer, MA, USA) every third day. Mice were euthanized 36 days following injection. The tumors were excised at the time of euthanasia. Immunohistochemistry was conducted to evaluate Notch3, PTEN, and Ki-67 expression. Tumor formation was determined by two independent pathologists. The above experiments were repeated three times independently with five nude mice in each group. In the three independent experiments, 15 nude mice in each group were used for data statistics. All the experiments were randomized.

### ONCOMINE analysis

The level of expression and the relationship between Notch3 and PTEN mRNA were searched and analyzed using the ONCOMINE (www.oncomine.org).

### GOBO analysis

The relationships between PTEN mRNA levels and different breast cancer subtype or ER status were analyzed from the GOBO database (http://co.bmc.lu.se/gobo/gsa.pl).

### Kaplan–Meier analysis

The Kaplan–Meier Plotter (http://kmplot.com) was used to analyze the correlation between Notch3 or PTEN mRNA expression and prognosis for recurrence-free and overall survival in breast cancer patients.

### Statistical analysis

SPSS 19.0 (SPSS Inc., Chicago, IL) was used for statistical analysis. The cell proliferation assay, migration/invasion assay, colony formation assay, and tumor xenograft models data were measured by using Student’s *t*-test. The rates were compared using Pearson’s chi-squared test. Spearman’s rank was used for correlation analysis. Survival curves were assessed using the Kaplan–Meier method and compared using the log-rank test from the database Kaplan–Meier Plotter. The statistical significance was defined as *P* < 0.05.

## Result

### Notch3 is expressed in the luminal subtype and modulates PTEN expression in breast cancer cell lines

**Fig. 1:**
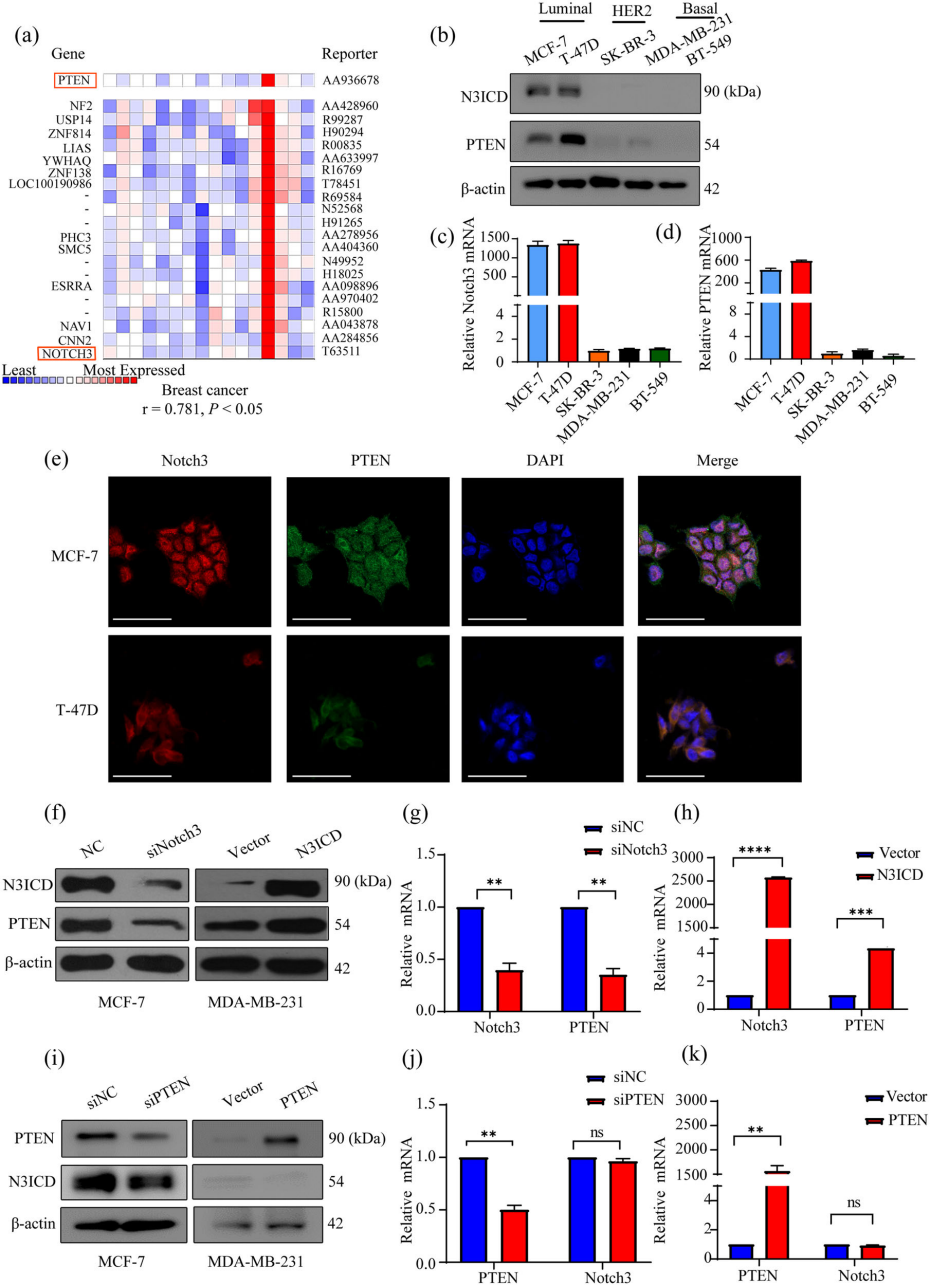
Notch3 is expressed in the luminal subtype and modulates PTEN expression in breast cancer cell lines. **a** Heat-map representing the correlation of the mRNA of PTEN, Notch3, and some other genes obtained from Weigelt Breast dataset based on the analyses of about 10,335 genes in ONCOMINE. There was a positive correlation between the mRNA level of Notch3 and PETN, the correlation coefficient *r* = 0.781, *P* < 0.05 (**b**) Notch3 and PTEN expression in distinct subtype of breast cancer cell lines detected by western blotting. **c**, **d** Relative Notch3 and PTEN mRNA levels in breast cancer cell lines quantified by real-time PCR. **e** The immunofluorescence staining of PTEN and Notch3 in MCF-7 and T-47D cells. Nuclei were counterstained with DAPI. **f**, **g** The levels of Notch3 and PTEN protein and mRNA in MCF-7 cells were detected by western blotting or real-time PCR, respectively, following Notch3 knockdown by siRNA. **f**, **h** Notch3 and PTEN protein and mRNA expression levels in MDA-MB-231 cells were measured by western blotting or real-time PCR, respectively, following expression of pCLE-N3ICD. **i**, **j** The levels of Notch3 and PTEN protein and mRNA in MCF-7 cells were detected by western blotting or real-time PCR, respectively, following PTEN knockdown by siRNA. **i**, **k** The levels of Notch3 and PTEN protein and mRNA in MDA-MB-231 cells were detected by western blotting or real-time PCR, respectively, following ectopic expression of PTEN in MDA-MB-231 cells. The experiments were repeated three times. The scale bar represents 50 μm. *P < 0.05, **P < 0.01, ***P < 0.001, ****P < 0.0001.

We initially conducted an extensive analysis of the ONCOMINE database to explore the relationship between Notch3 and PTEN. We found that Notch3 was highly correlated with PTEN at mRNA level (*r* = 0.781, *P* < 0.05) (Fig. 1a). To further investigate the role of Notch3 and PTEN in breast cancer, we evaluated their protein and mRNA expression levels in breast cancer cell lines representing distinct subtypes. Notch3 protein was primarily found in the luminal subtype (T-47D and MCF-7 cell lines) and virtually absent from the HER2-overexpressing cell line (SK-BR-3) and the TNBC cell lines (MDA-MB-231 and BT-549). Interestingly, PTEN expression paralleled Notch3 expression, with high levels observed in T-47D and MCF-7 cells and little to no expression in the SK-BR-3, MDA-MB-231, and BT-549 cell lines (Fig. 1b). Real-time PCR confirmed these differences in expression at the mRNA level (Fig. 1c, d). GOBO database analysis indicated that PTEN is highly expressed in luminal breast cancer cell lines (*P* = 0.0186), specifically in the luminal A patients (*P* < 0.00001) and ER-positive subtypes patients (*P* = 0.0106) (Fig. S1a–c).To further explore Notch3 and PTEN expression, we performed immunofluorescence staining for Notch3 and PTEN in MCF-7 and T-47D cells. As shown in Fig. 1e, Notch3 and PTEN co-localized in the luminal MCF-7 and T-47D breast cancer cell lines.

To determine the potential regulation between Notch3 and PTEN, we knocked down or overexpressed these two genes in breast cancer cells. Suppression of activated Notch3 (N3ICD) with siRNA significantly downregulated PTEN mRNA and protein expression in MCF-7 cells (Fig. 1f, g). In contrast, PTEN knockdown did not affect Notch3 expression (Fig. 1i, j). Conversely, Notch3ICD overexpression upregulated PTEN mRNA and protein levels in MDA-MB-231 cells (Fig. 1f, h), while PTEN overexpression did not alter Notch3 expression (Fig. 1i, k). These results suggest that Notch3 positively regulates PTEN expression by transcriptionally activating PTEN.

### Notch3 inhibits the AKT-mTOR pathway by activating PTEN via CSL-binding elements in the PTEN promoter

**Fig. 2:**
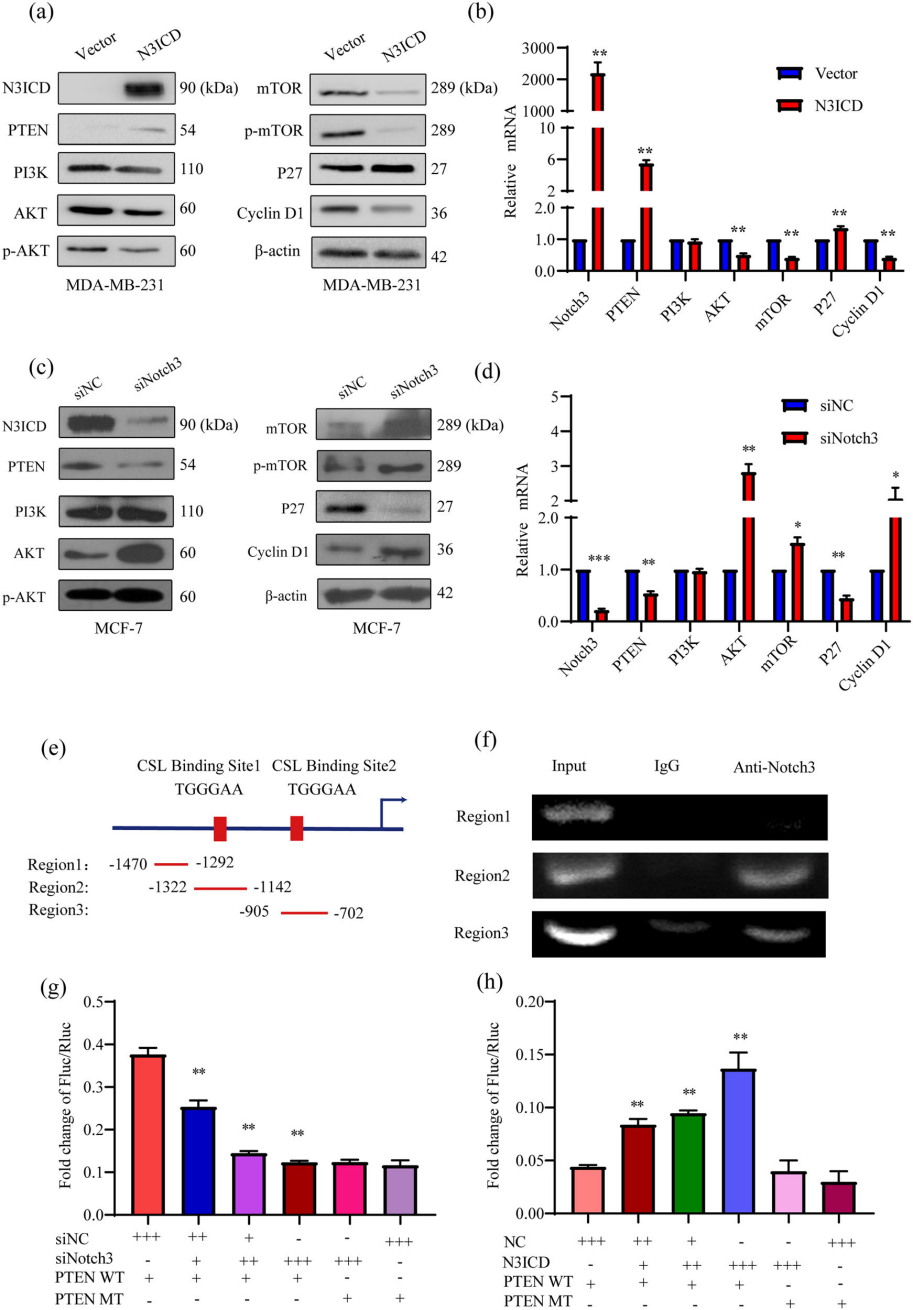
Notch3 inhibits the AKT-mTOR pathway by binding to the CSL element of the PTEN promoter. Notch3ICD overexpression increased PTEN and p27 expression in MDA-MB-231 cells but inhibited the protein (**a**) and mRNA (**b**) expression levels of AKT-mTOR-Cyclin D1 pathway components. Notch3 knockdown inhibited PTEN and p27 expression in MCF-7 cells but induced the protein (**c**) and mRNA (**d**) expression of AKT-mTOR-Cyclin D1pathway components. **e** Schematic diagram of the PTEN promoter regions amplified by different primer sets in the ChIP assays. Regions 2 and 3 each contain a CSL-binding element. Region 1, which does not contain the CSL-binding element, was used as a negative control. **f** For the ChIP assays, Notch3-binding PCR products were detected in regions 2 and 3 using an anti-Notch3 antibody. **g** Notch3 was knocked down in MCF-7 cells by siRNA, which were co-transfected with a wild-type or mutant PTEN promoter-containing firefly luciferase construct and pRL-SV40 as the control plasmid containing Renilla luciferase. The ratio of firefly luciferase to Renilla luciferase values was used to determine promoter activity. Each sample was performed in triplicate. **h** N3ICD overexpression in MDA-MB-231 cells co-transfected with a wild-type or mutant PTEN promoter-containing Firefly luciferase construct and pRL-SV40 as the control plasmid containing Renilla luciferase. The ratio of Firefly luciferase to Renilla luciferase was used to determine the promoter activity. Each sample was performed in triplicate. *P < 0.05, **P < 0.01.

To explore how Notch3 regulates PTEN expression and its effects on the downstream mTOR-AKT pathway, we measured AKT-mTOR pathway component expression levels following overexpression or silencing of Notch3. We found that ectopic expression of activated Notch3 in MDA-MB-231 cells induced expression of both PTEN and p27 but suppressed p-AKT and p-mTOR/mTOR levels, which paralleled changes in Cyclin D1 expression (Fig. 2a, b). Conversely, Notch3 knockdown in MCF-7 cells upregulated p-AKT and p-mTOR/mTOR levels while downregulating PTEN and p27 expression (Fig. 2c, d). These data support the hypothesis that Notch3 induces PTEN activity and inhibits the AKT-mTOR pathway, which plays a role in cell proliferation or the maintenance of the tumor suppressor phenotype in breast cancer.

Previous studies have clearly shown that the Notch family of transcription factors directly bind to CSL promoter elements to regulate downstream target molecules. Therefore, we explored whether Notch3 could regulate PTEN by directly binding to CSL elements in its promoter. For the chromatin immunoprecipitation (ChIP) assays, we designed two pairs of primers containing the core CSL binding sequence (TGGGAA) located upstream from the transcriptional start site (Region 2: –1322 to –1142 bp; Region 3: –905 bp to –702 bp) (Fig. 2e). As a negative control, we designed a third pair of primers to a region devoid of CSL-binding elements (Region1: –1470 bp to –1292 bp). Notch3 binding to the PTEN promoter in MCF-7 and MDA-MB-231 cells was observed by ChIP in the regions containing the CSL-binding elements; however, this binding was absent in the negative control (Fig. 2f). Subsequently, we evaluated PTEN transcriptional activity using a PTEN promoter-driven luciferase reporter in reporter to measure luciferase activity in the presence or absence of N3ICD. Following Notch3 silencing in MCF-7 cells, reporter activity decreased in a dose-dependent manner (*P* < 0.01). Conversely, the luciferase activity increased following N3ICD overexpression in MDA-MB-231 cells (*P* < 0.01). When comparing the activity of wild-type and mutant PTEN promoters (PTEN WT and PTEN MT, respectively), we noticed that N3ICD knockdown did not affect the luciferase activity of PTEN MT (Fig. 2g). Similarly, N3ICD overexpression had no impact on PTEN MT activity (Fig. 2h). These results suggest that Notch3 inactivating PTEN promoter via directly binding to the CSL binding elements present in the promoter.

### Ectopic Notch3 expression inhibits proliferation and migration/invasion in vitro, which is attenuated by PTEN silencing

**Fig. 3:**
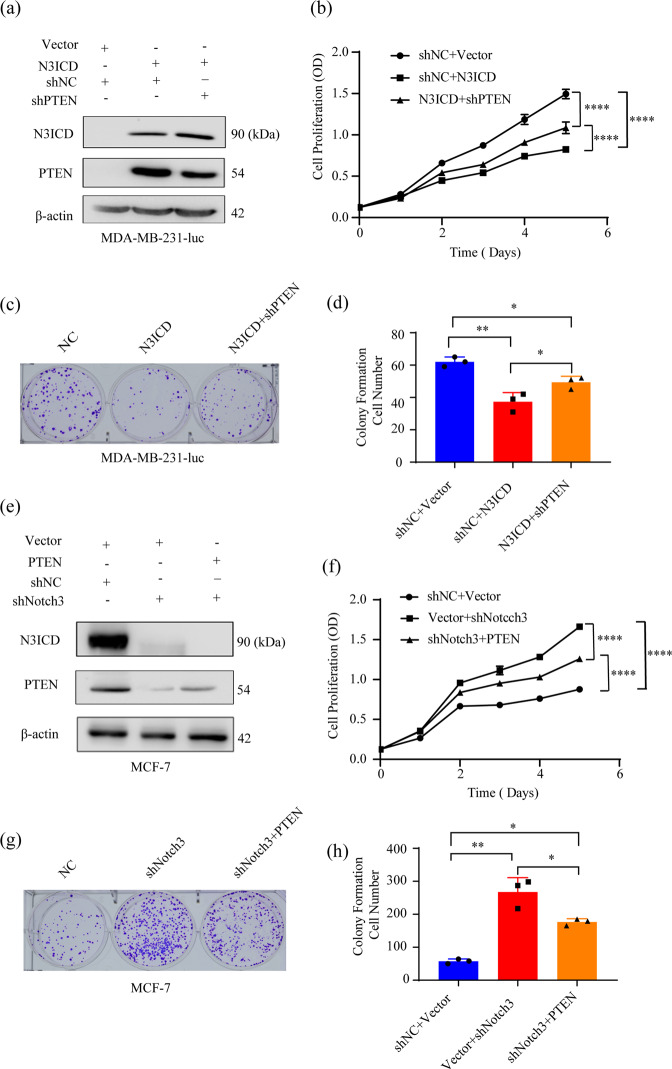
Ectopic Notch3 expression inhibits proliferation and tumorigenesis in vitro, which is attenuated by PTEN silencing. **a** Notch3 and PTEN expression in MDA-MB-231-luc cells measured by western blotting following stable N3ICD overexpression, with or without PTEN knockdown. β-actin served as an internal control. **b** Stable N3ICD expression inhibited MDA-MB-231-luc cell growth in vitro. This effect was attenuated by PTEN silencing with shRNA. **c** N3ICD overexpression inhibited colony formation, which was reversed by stable PTEN knockdown with shRNA. Representative pictures and quantitative data from the colony formation assays are presented. **d** Representative micrographs and data for Matrigel-coated assays. The stably transfected N3ICD MDA-MB-231-luc were co-transfected with shPTEN or shNC as a negative control. **e** Notch3 and PTEN expression in MCF-7 cells measured by western blotting following stable Notch3 knockdown, with or without stable PTEN overexpression. **f** Stable Notch3 knockdown induced MCF-7 cell growth in vitro, which was attenuated by PTEN overexpression. **g** Stable Notch3 knockdown promoted the colony formation, which was reversed by stable PTEN overexpression. Representative pictures and quantitative data from the colony formation assays are presented. **h** Representative micrographs for Matrigel-coated. Stably transfected shNotch3 MCF-7 cells were co-transfected with PTEN plasmid or negative control vector. Three independent experiments were performed, and all the data were analyzed using the two-sided *t*-test. *P < 0.05, **P < 0.01, ***P < 0.001, ****P < 0.0001.

The AKT-mTOR and Cyclin D1-P27 pathways play crucial roles in monitoring cell proliferation. Based on our results, we hypothesized that the Notch3-PTEN axis modulates cell proliferation and tumorigenesis. To assess the ability of the Notch3-PTEN axis to regulate cell proliferation, we examined cell proliferation after stable Notch3ICD knockdown or overexpression in MCF-7 and MDA-MB-231-luc cells, respectively, with or without enforced PTEN expression. Changes in Notch3 and PTEN protein levels were detected by western blotting (Fig. 3a, e). The proliferation of MDA-MB-231-luc cells overexpressing Notch3 decreased by 37.7% after 3 days and 44.8% after 5 days, Silencing of PTEN expression in these cells reversed this phenomenon (*P* < 0.0001) (Fig. 3b). In contrast, the proliferation of Notch3-silenced MCF-7 cells increased 1.69-fold and 1.90-fold after 3 and 5 days, respectively. This effect was reversed by ectopic PTEN expression (*P* < 0.0001) (Fig. 3f). We also evaluated the effects of modulating Notch3 and PTEN expression on breast cancer cell proliferation using the colony formation assay. MDA-MB-231-luc cells stably overexpressing activated Notch3 had a reduced ability to form colonies (39.9% decrease after 2 weeks) (Fig. 3c, d). This phenotype was reversed by knocking down PTEN expression with shRNA (*P* < 0.05). Conversely, Notch3 knockdown in MCF-7 cells increased colony formation 1.60-fold after 2 weeks, which was reversed by PTEN overexpression (*P* < 0.05) (Fig. 3g, h).

We further assessed the role of Notch3 in MDA-MB-231-luc cells and MCF-7 cells using migration and invasion assays. Overexpression of activated Notch3 decreased cell migration by nearly 70%, which was partially rescued by PTEN silencing (*P* < 0.01) (Fig. S2a, b). In contrast, Notch3 knockdown increased cell migration 10-fold, which was partially abrogated by PTEN overexpression (*P* < 0.05) (Fig. S2d, e). Similar results were obtained with the in vitro invasion assays (Fig. S2a, b, c, f). Moreover, there was no effect on cell death as measured by TUNEL staining in ectopic Notch3ICD expression cell line. These data indicate that Notch3 induces PTEN expression, which subsequently inhibits proliferation and migration/invasion, an effect that can be attenuated by silencing PTEN in vitro.

### Notch3 inhibits the growth of breast cancer xenografts by regulating PTEN

**Fig. 4:**
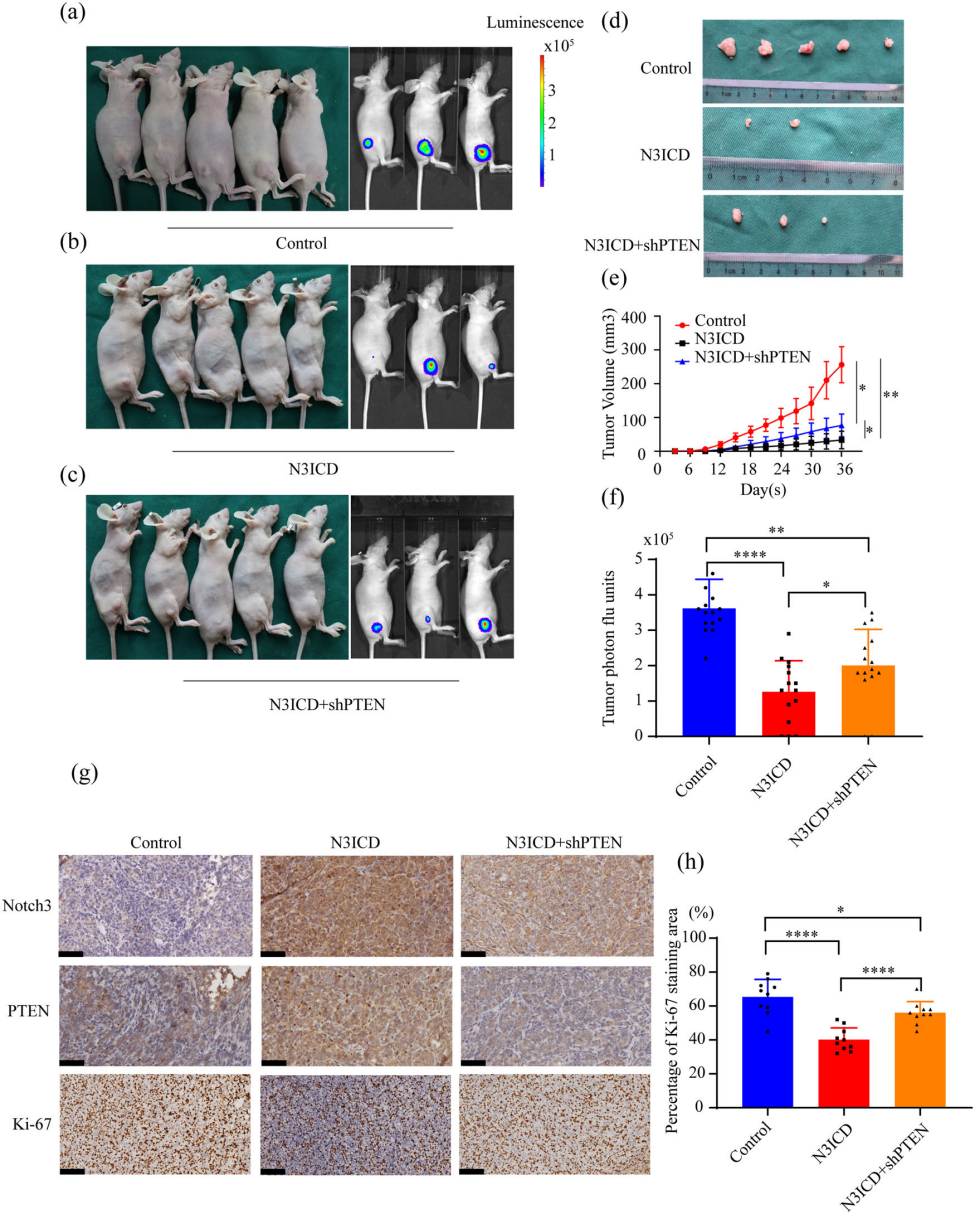
Notch3 inhibits cell proliferation by regulating PTEN in vivo. **a**–**c** Control vector, Notch3-expressing, and N3ICD + shPTEN MDA-MB-231-Luc cells (2 × 10^6^ cells) were injected subcutaneously into the right thigh of immunodeficient NU/NU mice (*n* = 5). Tumor size was evaluated every three days. **d** Primary tumors from mice inoculated with vector, N3ICD, or N3ICD + shPTEN transfected MDA-MB-231-luc cells were collected 36 days post-inoculation. **e** Time course of tumor growth is shown. **f** Quantitation of bioluminescence using the IVIS Kinetic Imaging System. **g** Immunohistochemical staining for Notch3, PTEN, and Ki-67 was performed on dissected tumors. **h** Quantitative analysis of Ki-67 staining was performed. Original magnification, 200x. The scale bar indicates 50 μm. *P < 0.05, **P < 0.01, ***P < 0.001, ****P < 0.0001.

To explore the effect of Notch3 on tumorigenesis, we examined the effects of modulating the Notch3-PTEN axis on the growth of MDA-MB-231-luc-N3ICD tumor cells in nude mice. The subcutaneous tumors were followed either by caliper measurement or bioluminescence imaging (BLI). Five weeks after inoculation, mice implanted with MDA-MB-231-luc-N3ICD cells developed fewer than those inoculated with MDA-MB-231-luc-NC cells (2/5 vs. 5/5). The size of the tumors was also reduced. These effects were reversed by PTEN overexpression (Fig. 4a–e). Similarly, BLI showed that mice inoculated with MDA-MB-231-luc-N3ICD tumor cells consistently had an ≈3-fold decrease in tumor burden compared to mice inoculated with control cells (Fig. 4f). Immunohistochemistry of tumors harvested from mice at the end of the experiment showed that tumors derived from control cells lacking N3ICD expressed higher levels of the proliferation index marker Ki-67 than the other tumor types. Consistent with the in vitro data, tumors derived from Notch3-overexpressing cells expressed higher levels of Notch3 and PTEN. In addition, tumors derived from N3ICD-overexpressing cells with PTEN knockdown expressed medium intensity Ki-67, confirming that Notch3-mediated PTEN regulation was retained in vivo (Fig. 4g, h). These results demonstrated that Notch3 could inhibit the proliferation of breast cancer in vivo by modulating PTEN levels.

### Notch3 and PTEN expression positivity correlate in breast cancer patients

**Fig. 5:**
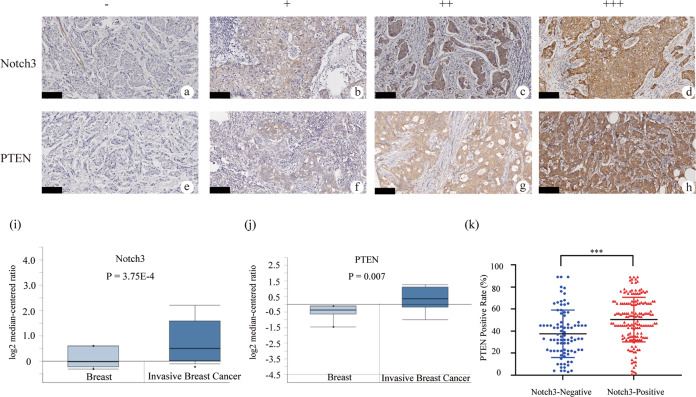
Notch3 expression is associated with PTEN in clinical breast cancer specimens. **a** Representative image of Notch3-negative (−) (**b**) Notch3-weak positive (+) (**c**) Notch3-medium positive (++) (**d**) Notch3-strong positive (+++) (**e**) Representative image of PTEN-negative (−) (**f**) PTEN-weak positive (+) (**g**) PTEN-medium positive (++) (**h**) PTEN-strong positive (+++) specimens, original magnification, 400x. Comparison of the mRNA level of (**i**) Notch3 and (**j**) PTEN in Finak Breast dataset by ONCOMINE analysis. **k** The positive correlation between Notch3 and PTEN expression was determined using the two-sided *t*-test. The scale bar for 100 μm. *** *P* < 0.001.

Accumulating evidence demonstrated that Notch3 and ER were highly correlated in different subtypes of breast cancer, which was associated with a better prognosis^32^. Moreover, previous research revealed an inverse relationship between the risk of death and PTEN expression. Therefore, we evaluated the relationships between Notch3 and PTEN expression and the pathological parameters of 250 human invasive ductal carcinoma tissue samples. Positive Notch3 expression was found in 162 of the 250 specimens (64.8%) (Fig. 5b–d) and the Notch3-negative shown in Fig. 5a. PTEN expression was positive in 172 of the 250 samples (68.8%) (Fig. 5f–h) and the PTEN-negative shown in Fig. 5e. The different expression levels of Notch3 and PTEN shown in Fig. 5a–h.

The relationships between Notch3 and PTEN expression and the pathological features of breast cancer cases are presented in Table 1. A significant positive relationship between Notch3 or PTEN expression and lower N3 lymph node involvement was observed. Notch3-positive patients were significantly higher in N0 (69.6%) and N1 groups (71.4%) compared to the N2 (54.3%) and N3 (45.5%) groups (*P* = 0.020). PTEN-positive patients were higher in N0 (79.3%) and N1 groups (74.0%) compared to the N2 (55.7%) and N3 (27.3%) groups (*P* < 0.001). As shown in Table 1, Notch3 was positively related to the estrogen receptor (ER), progesterone receptor (PR), lower incidence of nodal involvement, and lower Ki-67 index. Of the 162 patients, the Notch3 positivity rate was significantly higher in ER+ (112/162; 69.1%) than ER− (50/162; 30.9%) breast cancer (*P* = 0.020). It was also higher in PR+ (104/162; 64.2%) than PR− (58/162; 36.3%) breast cancer (*P* = 0.044). Thus, Notch3 expression was positively correlated with both ER and PR expression. Similarly, a relationship was observed between PTEN and the estrogen receptor (ER), Ki67 expression, and nodal status. Out of 172 PTEN-positive patients, the PTEN positivity rate was higher in ER+ (125/172; 72.7%) than ER− tumors (*P* < 0.001). Patients in the Ki-67-negative group were also more likely to be defined as Notch3-positive (Notch3-positive frequency: 80/108; 74.1%, *P* = 0.007). Similarly, a higher PTEN positivity rate was observed with the Ki-67-negative group than the Ki-67-positive group (PTEN-positive frequency: 92/122; 75.4%, *P* = 0.030). In contrast, there was no significant difference in age, menopausal status, histological grade, and molecular subtype between different expression levels of Notch3 or PTEN. ONCOMINE analysis revealed that Notch3 and PTEN mRNA expression levels were both significantly higher in invasive breast cancer than in normal tissue in the Finak Breast dataset. In particular, Notch3 expression was elevated 1.741-fold in breast cancer compared to normal tissue (*P* = 3.75E-4), and PTEN was elevated 1.702-fold (*P* = 0.007) (Fig. 5i, j). In this cohort, we observed a positive correlation between Notch3 and PTEN expression (*P* < 0.001) (Fig. 5k). Taken together, these data indicated that Notch3 expression is positively correlated with elevated PTEN expression and associated with a lower capacity of tumor proliferation and incidence of involved node status.

**Table 1:** Association between clinicopathological characteristics and Notch3 or PTEN expression in breast cancer patients.

Features	Notch3 (%)			PTEN (%)		
	Negative (*N* = 88)	Positive (*N* = 162)	*P*	Negative (*N* = 78)	Positive (*N* = 172)	*P*
Age						
≤50	35 (31.0)	78 (69.0)	0.204	30 (26.5)	83 (73.5)	0.149
>50	53 (38.7)	84 (61.3)		48 (35.0)	89 (65.0)	
Menopausal status						
Pre	34 (31.8)	73 (68.2)	0.327	33 (30.8)	74 (69.2)	0.916
Post	54 (37.8)	89 (62.2)		45 (31.5)	98 (68.5)	
Tumor size (cm)						
≤2	35 (44.3)	44 (55.7)	0.120	20 (25.3)	59 (74.7)	0.058
>2, ≤5	35 (31.5)	76 (68.5)		32 (28.8)	79 (71.2)	
>5	18 (30.0)	42 (70.0)		26 (43.3)	34 (56.7)	
Nodal status						
N0	28 (30.4)	64 (69.6)	**0.020**	19 (20.7)	73 (79.3)	**<0.001**
N1	22 (28.6)	55 (71.4)		20 (26.0)	57 (74.0)	
N2	32 (45.7)	38 (54.3)		31 (44.3)	39 (55.7)	
N3	6 (54.5)	5 (45.5)		8 (72.7)	3 (27.3)	
Histological grade						
I	10 (35.7)	18 (64.3)	0.539	7 (25.0)	21 (75.0)	0.240
II	45 (32.8)	92 (67.2)		35 (25.5)	102 (74.5)	
III	33 (38.8)	52 (61.2)		36 (42.4)	49 (57.6)	
Ki67						
Negative (≤20%)	28 (25.9)	80 (74.1)	**0.007**	30 (24.6)	92 (75.4)	**0.030**
Positive (>20%)	60 (42.2)	82 (57.8)		48 (37.5)	80 (62.5)	
ER						
Negative	40 (32.0)	50 (68.0)	**0.020**	43 (47.8)	47 (52.2)	<**0.001**
Positive	48 (44.4)	112 (55.6)		35 (21.9)	125 (78.1)	
PR						
Negative	43 (42.6)	58 (57.4)	**0.044**	30 (27.0)	81 (73.0)	0.219
Positive	45 (30.2)	104 (69.8)		48 (34.5)	91 (65.5)	
HER2						
Negative	60 (34.3)	115 (65.7)	0.644	49 (28.0)	126 (72.0)	0.095
Positive	28 (37.3)	47 (62.7)		29 (38.7)	46 (61.3)	
Molecular subtypes						
Luminal A	15 (50.0)	15 (50.0)	0.233	12 (40.0)	18 (60.0)	0.098
Luminal B	32 (33.7)	63 (66.3)		30 (31.6)	65 (68.4)	
HER2-enriched	22 (29.3)	53 (70.7)		16 (21.3)	59 (78.7)	
TNBC	19 (38.0)	31 (62.0)		20 (40.0)	30 (60.0)	

Statistically significant (*P* < 0.05) values are in bold.

### High expression both of Notch3 and PTEN mRNA predicts better prognostic in breast cancer patients

**Fig. 6:**
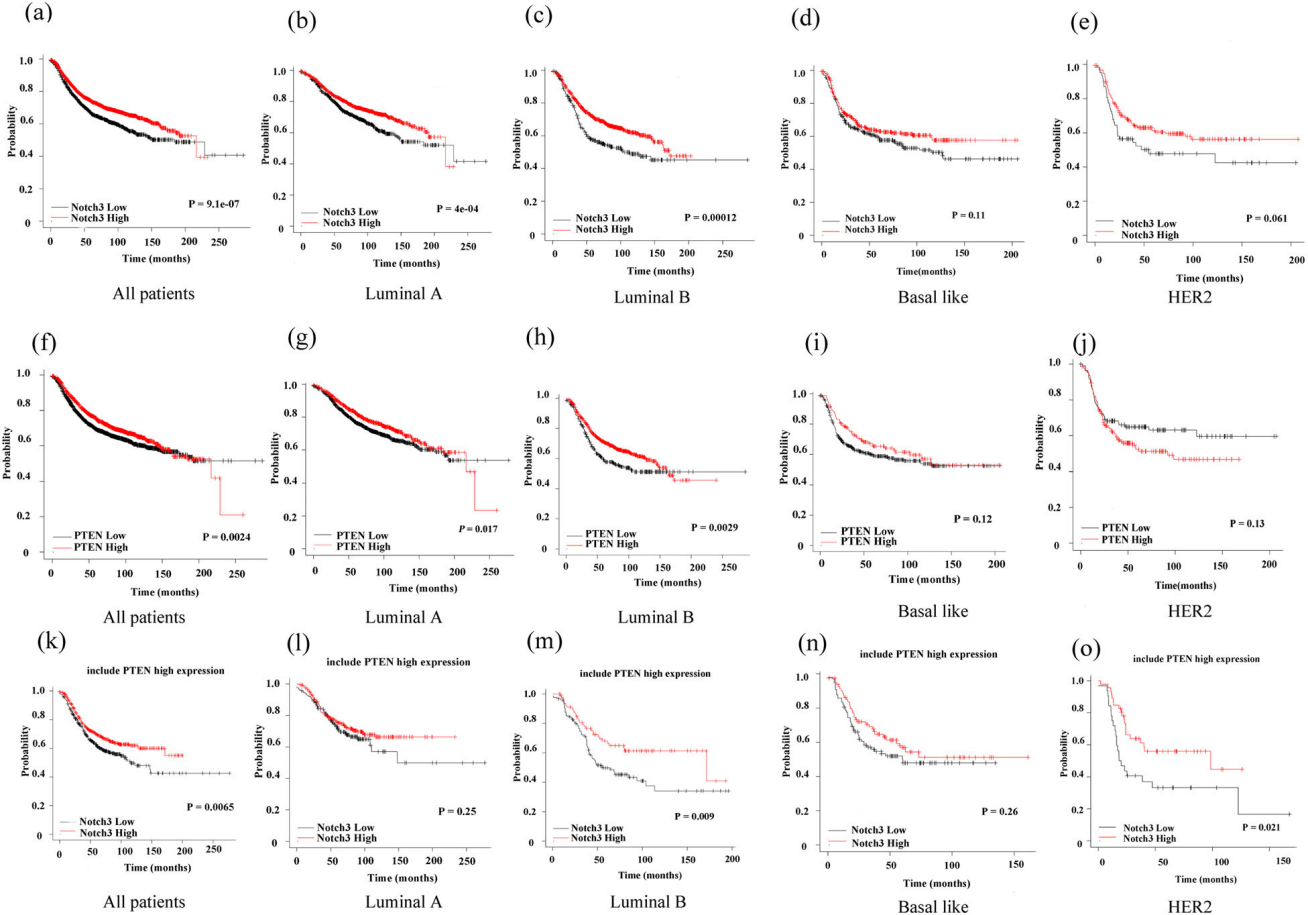
High Notch3 and PTEN mRNA expression predicts a better RFS in breast cancer patients. High expression of Notch3 (**a**–**e**) or PTEN (**f**–**h**) resulted in a better RFS among different breast cancer subtypes. PTEN levels had no effect on RFS among the basal-like (**i**) and HER2-positive (**j**) subtypes. A better RFS was observed for breast cancer patients expressing both high Notch3 and high PTEN levels with all patients, luminal B or HER2-positive subtypes (**k**, **m**, **o**) but not the luminal A or basal-like subtype (**l**, **n**).

The prognosis of Notch3- and PTEN-positive breast cancer patients across different subtypes was estimated from the Kaplan–Meier Plotter clinical database. We found that patients with elevated Notch3 expression had a better relapse-free survival (RFS) rate (*P* = 9.1 e-07) (Fig. 6a). Those patients with high PTEN expression levels also had better RFS (*P* = 0.0024) (Fig. 6f). After restricting the analysis to intrinsic molecular breast cancer subtypes, we found that high Notch3 expression had a better prognosis for those with the luminal A, luminal B subtype compared to those with low Notch3 expression (Fig. 6b, c). Moreover, patients with luminal A or luminal B breast cancer tended to have a better RFS if PTEN was overexpressed (Fig. 6g, h). Notably, high mRNA expression of both Notch3 and PTEN predicted better relapse-free survival in overall breast cancer patients (*P* = 0.0065) (Fig. 6k). Similarly, this expression pattern revealed a good prognosis for OS in those patients with Notch3 high expression including all patients, luminal A, and basal-like subtype. PTEN expression levels also had better OS in luminal A subtype (Fig. S3). In conclusion, Notch3 and PTEN mRNA overexpression were indicative of a good prognosis for breast cancer patients.

## Discussion

The Notch receptor is a highly conserved type I transmembrane glycoprotein involved in cell differentiation, proliferation, and cell survival and has an important role in numerous tumors. The Notch3 gene was originally described as being expressed in proliferative neuroepithelium^34^. Although its basic structure is similar to Notch1 and Notch2, Notch3 has many structural differences, including a shorter TAD (transcription activation domain) domain and differences in the intracellular domain^35^. Among the four mammalian Notch proteins, Notch3 has a more limited tissue distribution, mainly restricted to vascular smooth muscle, the central nervous system, and certain thymocyte subsets. Increasing evidence suggests that Notch3 may play a significant role in breast development and maintenance of the luminal fate^36^. Indeed, our previous research in breast cancer established that Notch3 maintains the luminal phenotype and inhibits tumor formation and metastasis via activating ERα^32^. Our current study firstly demonstrates that Notch3 transcriptionally upregulates PTEN and its downstream pathway by binding to the CSL elements in the PTEN promoter, and leads to the inhibition of breast cancer cell proliferation and migration/invasion. Furthermore, we showed that with high expression of Notch3 and PTEN had better RFS in breast cancer patients.

PTEN, the most commonly mutated cancer gene^37^, is located at 10q23.31with nine exons that encode a molecule of 403 amino acids. Saala et al.^38^ showed that abnormal PI3K/PTEN signaling was closely associated with metastasis and low survival rates among different tumor types, emphasizing the potential impact of pathway inhibition on patient survival. PTEN dephosphorylates ERK1/2 phosphatase in the nucleus, leading to MAPK pathway activation and cyclin D1 downregulation^39^. PTEN activity is lost due to protein, genetic, or epigenetic changes has been observed in nearly half of breast cancer cases^40^. Accumulating evidence has shown that the Notch pathway regulates PTEN in various cancers, such as T-cell leukemia^41^ and prostate cancer^42^. Baker et al.^5^ demonstrated that Notch1-mediated suppression of PTEN was necessary for proliferation and stem cell survival. Of note, Sizemore et al.^43^ demonstrated that mammary epithelial stem cell (MaSC) enriched from PTEN-null mice had improved stem cell activity in vitro, and the loss of PTEN regulated the precancerous breast stem cell niche by “altering” the paracrine signal from Jagged-1 to Notch3.

The results of this study indicated that PTEN was highly expressed in MCF-7 and T-47D, classified as luminal-type breast cancer cell lines but expressed at low levels in the HER2-overexpressing subtype (SK-BR-3), TNBC (MDA-MB-231), and PTEN-deficient breast cancer (BT-549). ONCOMINE database analysis indicated that PTEN was highly expressed in luminal and ER-positive breast cancer. Interestingly, the level of Notch3 mRNA and protein levels were higher in luminal-type (ER-positive) breast cancer and lower in ER-negative breast cancer. Our investigation of the regulatory mechanism revealed that Notch3 suppression significantly downregulated the levels of PTEN mRNA and protein in MCF-7 cells. Conversely, ectopic Notch3ICD expression upregulated PTEN in MDA-MB-231 cells. Intriguingly, Whelan et al.^44^ found that Notch1 was involved in the regulation of PTEN by binding to CBF-1. CBF-1 is one of the transcriptional factors for Notch1 activation, which combines the core DNA sequence C/G TGGGAA A/C. As a transcriptional inhibitor, CBF-1 forms a repressor complex with Skip, SMRT/N-CoR, and HDAC^45^. Moreover, our previous studies demonstrated that the CSL transcription complex element GGGAA was involved in the regulation of downstream genes, including ER and GATA3^32,46^. Mechanically, we showed that Notch3 binds to PTEN promoter which contains CSL-binding elements. In the current study, we found that after Notch3 silencing in MCF-7 cells, the PTEN-mediated luciferase reporter activity reduced in a concentration-dependent manner. Conversely, the PTEN-mediated luciferase activity increased following the overexpression of N3ICD in MDA-MB-231 cells. Other studies have shown that the other two transcription factors (BMI1 and c­JUN) are dysregulated in a variety of cancers and inhibit PTEN transcription^47,48^.

**Fig. 7:**
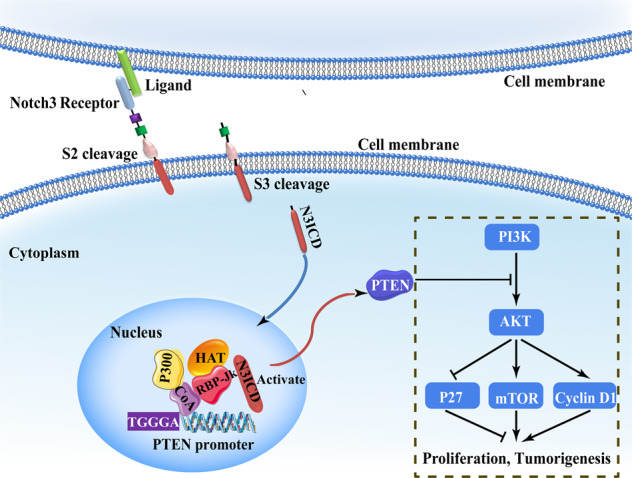
A hypothetical model for the regulation of cell proliferation and tumorigenesis by the Notch3ICD/PTEN axis. Notch3 is subjected to S2 and S3 cleavage, which generates Notch3ICD. Notch3ICD binds directly to the CSL-binding elements in the PTEN promoter, resulting in transactivation of PTEN and its downstream signaling pathway and inhibition of breast cancer cell proliferation and tumorigenesis.

Different Notch receptors may have opposite effects on proliferation in mammals. Lafkas et al.^15^ reported that Notch3 could inhibit proliferation in the mammary gland. Higher levels of Notch1 can also cause growth suppression in MCF-10A cells, whereas active Notch1 can promote proliferation in the same tissue, albeit in different cell subpopulations^49^. PTEN acts as a negative regulatory factor of the PI3K- Akt pathway. It is one of the most interesting tumor suppressor factor involved in cell growth, survival, migration and genomic stability. Based on our previous data and current results, we investigated whether Notch3 and PTEN inhibited the ability of breast cancer cells to proliferate and found that activated Notch3 induced PTEN in vitro, and high Notch3 and PTEN expression levels were associated with a weaker ability to proliferate, suggesting that these pathways, at least in part, may be involved in the inhibition of tumorigenesis. Chung et al.^39^ revealed that cytoplasmic PTEN acts as a lipid phosphatase and dephosphorylates PIP3, leading to attenuation of activated Akt levels and upregulation of the cell cycle inhibitor p27 and proapoptotic caspase3/7, which, in turn, induces apoptosis. In this study, we found that ectopic expression of Notch3ICD in MDA-MB-231 induced PTEN and p27 expression but suppressed p-AKT and p-mTOR/mTOR, which paralleled the changes in CyclinD1 levels. More importantly, Chen et al.^17^ demonstrated that Notch3 upregulated Cdh1 and induced p27 accumulation by impacting Skp2 degradation, leading to cell cycle arrest and inhibition of breast cancer cell proliferation. Consistent with this result, we demonstrated that Notch3ICD knockdown promoted MCF-7 cell proliferation, while forced expression of Notch3ICD caused inhibition of proliferation in vitro. Similarly, the mediating effect of Notch3 in PTEN signaling was also found in in vivo experiments. We demonstrated that Notch3ICD overexpression in xenografts significantly reduced tumor size than the control group. While PTEN knockdown significantly reversed the inhibitory effect of Notch3 on tumor proliferation in the N3ICD + shPTEN model. In addition, immunohistochemical staining showed that N3ICD induced PTEN expression and inhibited Ki-67 in N3ICD xenograft tumor model, at the same time, PTEN knockdown resulted in loss of PTEN expression and increased Ki-67 staining in the N3ICD + shPTEN group, suggesting that Notch3 overexpression may inhibit tumor formation by regulating PTEN expression. These data confirmed our hypothesis that activated Notch3 could induce PTEN expression and inhibit the AKT-mTOR pathway, thus inhibiting proliferation/migration or maintaining the tumor suppressor phenotype, as illustrated in Fig. 7. Future experiments will verify whether Notch3 can activate PTEN in PTEN-deficient breast cancer cell lines.

Our previous research indicated that expression of Notch3 positively correlates with ER, PR, and GATA-3 expression, a lower incidence of lymph node involvement, and better prognosis^32,46^. In the study, we found that Notch3 positively correlated with PTEN, consistent with the abundance of ER and PR. In contrast, Notch3 negatively correlated with Ki-67 expression and lymph node status. Ki-67, a proliferation index marker, acts as a predictor of tumor proliferation, with the worst breast cancer prognosis associated with a high Ki-67 index. Our study found that Notch3-PTEN was negatively correlated with Ki-67 proliferation index. In order to verify whether Notch3-PTEN high expression could predict the prognosis and survival of breast cancer patients, we analyzed the prognosis of patients with high or low Notch3 or PTEN mRNA expression from the open database, Kaplan-Meier Plotter. Intriguingly, previous studies have confirmed that Notch3 or PTEN overexpression indicated a better prognosis^32,40^. Moreover, the mRNA expression of both Notch3 and PTEN predicted a better RFS for overall breast cancer patients. This finding is supported by the work of Sizemore et al., who demonstrated that low stromal expression of the Notch ligand JAG1 and PTEN correlated with worse prognosis in breast cancer patients^43^.

In general, we have identified a positive correlation between Notch3 and PTEN in breast cancer and confirmed a role for the Notch3-PTEN axis in inhibiting tumor proliferation and migration/invasion. In addition, we explored in detail and first clarified the possible molecular mechanism of regulating this axis. Our results provide new insights into the complex regulation of breast cancer proliferation and tumorigenesis. We believe that the Notch3-PTEN axis may be a prospective prognostic predictor for breast cancer.

## Supplementary information

Supplementary-Table

F-Fig.S1

F-Fig.S2

F-Fig.S3

Supplementary figure legend
