# Supplementary material for: Notch3 inhibits cell proliferation and tumorigenesis and predicts better prognosis in breast cancer through transactivating PTEN
Source: Cell Death Dis. 2021 May 18;12(6):502. doi: 10.1038/s41419-021-03735-3 (PMC8131382; doi:10.1038/s41419-021-03735-3)
Supplement: Supplementary file 1 — Supplementary-Table [file 41419_2021_3735_MOESM1_ESM.docx]

**Table S1. Antibodies used in the study**

| Antibody | Company | Cat. Number | Dilution |
| --- | --- | --- | --- |
| Notch3  PTEN  PI3K(p110α)  AKT  p-AKT(Ser473)  mTOR  p-mTOR(Ser2448)  p27  CyclinD1  β-actin  Notch3*#  PTEN*#  ER#  PR#  HER2#  Ki67#  anti-rabbit IgG*#  anti-mouse IgG*# | CST, USA  CST, USA  CST, USA  CST, USA  CST, USA  CST, USA  CST, USA  CST, USA  CST, USA  Santa Cruz, USA  abcam, USA  abcam, USA  MXB, China  MXB, China  CST, USA  MXB, China  CST, USA  CST, USA | 5276T  9188T  4249T  4691T  4060T  2983T  5536T  3686T  55506T  sc-8432  ab23426  ab170941  MAB-0062  MAB-0675  4290S  MAB-0672  7074S  7076S | 1:2000  1:2000  1:2000  1:2000  1:2000  1:2000  1:2000  1:2000  1:2000  1:1000  1:400  1:400  1:400  1:400  1:400  1:400  1:3000  1:3000 |

*Used for immunofluorescence, #Used for immunohistochemistry

**Table S2. The target sequences of small interfering RNA (siRNA) or short hairpin RNA (shRNA) used in the study**

| Name | Target Sequences |
| --- | --- |
| Scramble Sense  Scramble Antisense  Notch3 Sense  Notch3 Antisense  PTEN Sense  PTEN Antisense  pGPU6/RFP/Neo/shNotch3 target sequence*  pGPU6/RFP/Neo/shPTEN target sequence*  pGPU6/RFP/Neo/shNC target sequence* | UUCUCCGAACGUGUCACGUTT  ACGUGACACGUUCGGAGAATT  UAUAGGUGUUGACGCCAUCCACGCA  UGCGUGGAUGGCGUCAACACCUAUA  GUUAGCAGAAACAAAAGGAGAUAUCAA  UUGAUAUCUCCUUUUGUUUCUGCUAAC  GAGCCAATAAGGACATGCA  GTTAGCAGAAACAAAAGGAGATATCAA  GTTCTCCGAACGTGTCACGT |

*shRNA plasmid

**Table S3. PCR or Quantitative Real-Time PCR primers used in the study**

| Name | Sequences of Primer |
| --- | --- |
| Notch3 Forward Primer  Notch3 Reverse Primer  PTEN Forward Primer  PTEN Reverse Primer  β-actin Forward Primer  β-actin Reverse Primer  PI3K(PIK3CA) Forward Primer  PI3K(PIK3CA) Reverse Primer  AKT(AKT1) Forward Primer  AKT(AKT1) Reverse Primer  mTOR (mTORC1) Forward Primer  mTOR (mTORC1) Reverse Primer  p27 Forward Primer  p27 Reverse Primer  Cyclin D1 Forward Primer  Cyclin D1Reverse Primer  PTEN wild Forward Primer-1*  PTEN wild Reverse Primer-1*  PTEN wild Forward Primer-2*  PTEN wild Reverse Primer-2*  PTEN mutant Forward Primer*  PTEN mutant Reverse Primer* | ATGCAGGATAGCAAGGAGGA  AAGTGGTCCAACAGCAGCTT  AAAGGCACAAGAGGCCCTAGAT  CAAGTTCCGCCACTGAACATTGGAA  CATGGAGTCCTGTGGCATCC  AATGCCAGGGTACATGGTGG  TGGATGCTCTACAGGGCTTT  GTCTGGGTTCTCCCAATTCA  TCTATGGCGCTGAGATTGTG  CTTAATGTGCCCGTCCTTGT  AGGCCGCATTGTCTCTATCAA  GCAGTAAATGCAGGTAGTCATCCA  GGGGCTCGTCTTTTCGGGGTGTTT  GAGCGGGAGGGCGGAGAGGAG  AACTACCTGGACCGCTTCCT  CCACTTGAGCTTGTTCACCA  CGAGCTCCCCGAGCAAAGGAAGAAGAC  TCCCCCGGGTACGGAACGGTAGGAAGCTG  CGAGCTCGCAGCAGGAGATACCCTCAA  TCCCCCGGGAAGAGCAAGCCAAAGGACTG  GCTATCACTGGGGAGTACTAATTTGGAAAGTTCC  GGAACTTTCCAAATTAGTACTCCCCAGTGATAGC |

*PCR Primer
