# Supplementary figures and images for: Notch3 inhibits cell proliferation and tumorigenesis and predicts better prognosis in breast cancer through transactivating PTEN
Source: Cell Death Dis. 2021 May 18;12(6):502. doi: 10.1038/s41419-021-03735-3 (PMC8131382; doi:10.1038/s41419-021-03735-3)

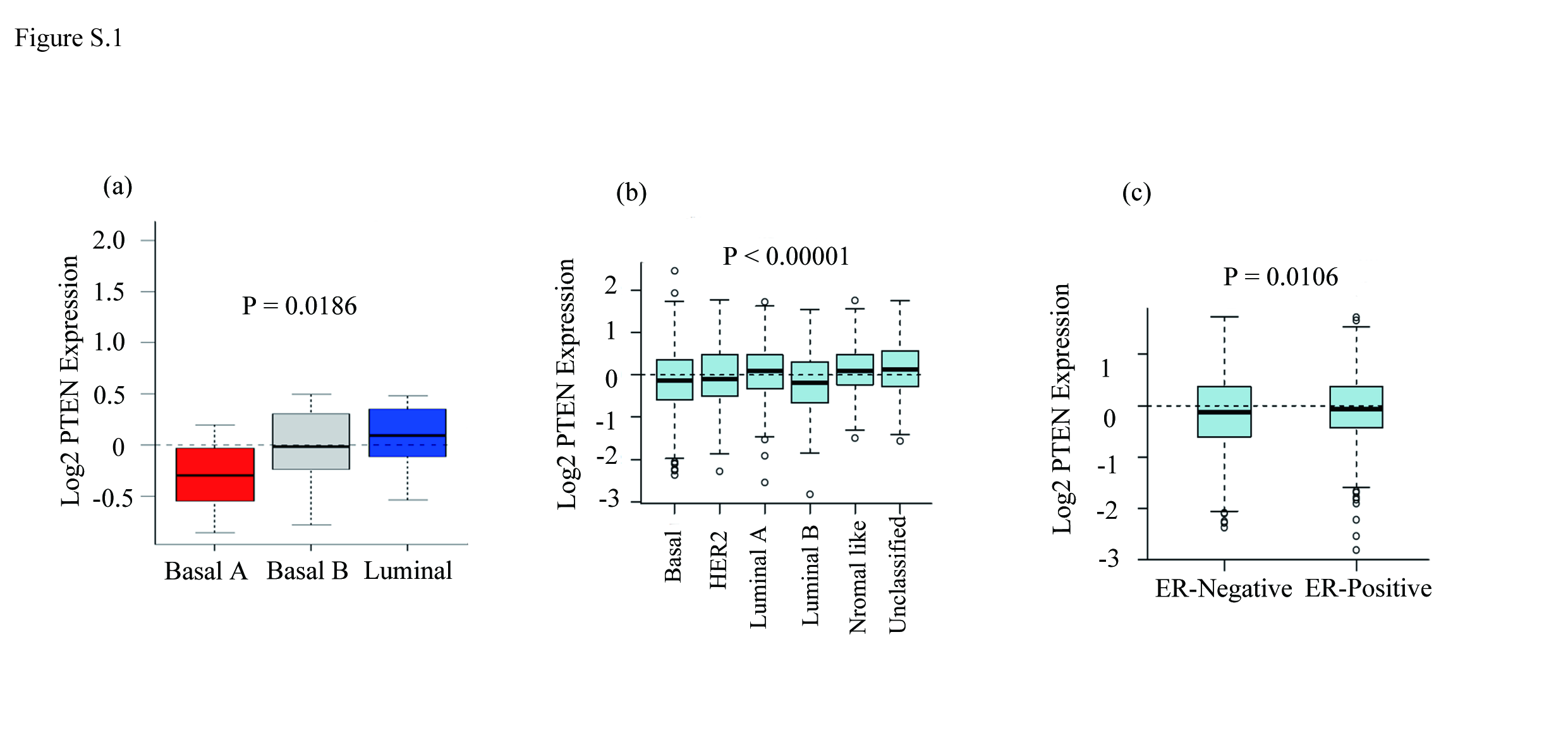

Supplement: Supplementary file 2 — F-Fig.S1 [file 41419_2021_3735_MOESM2_ESM.tif]

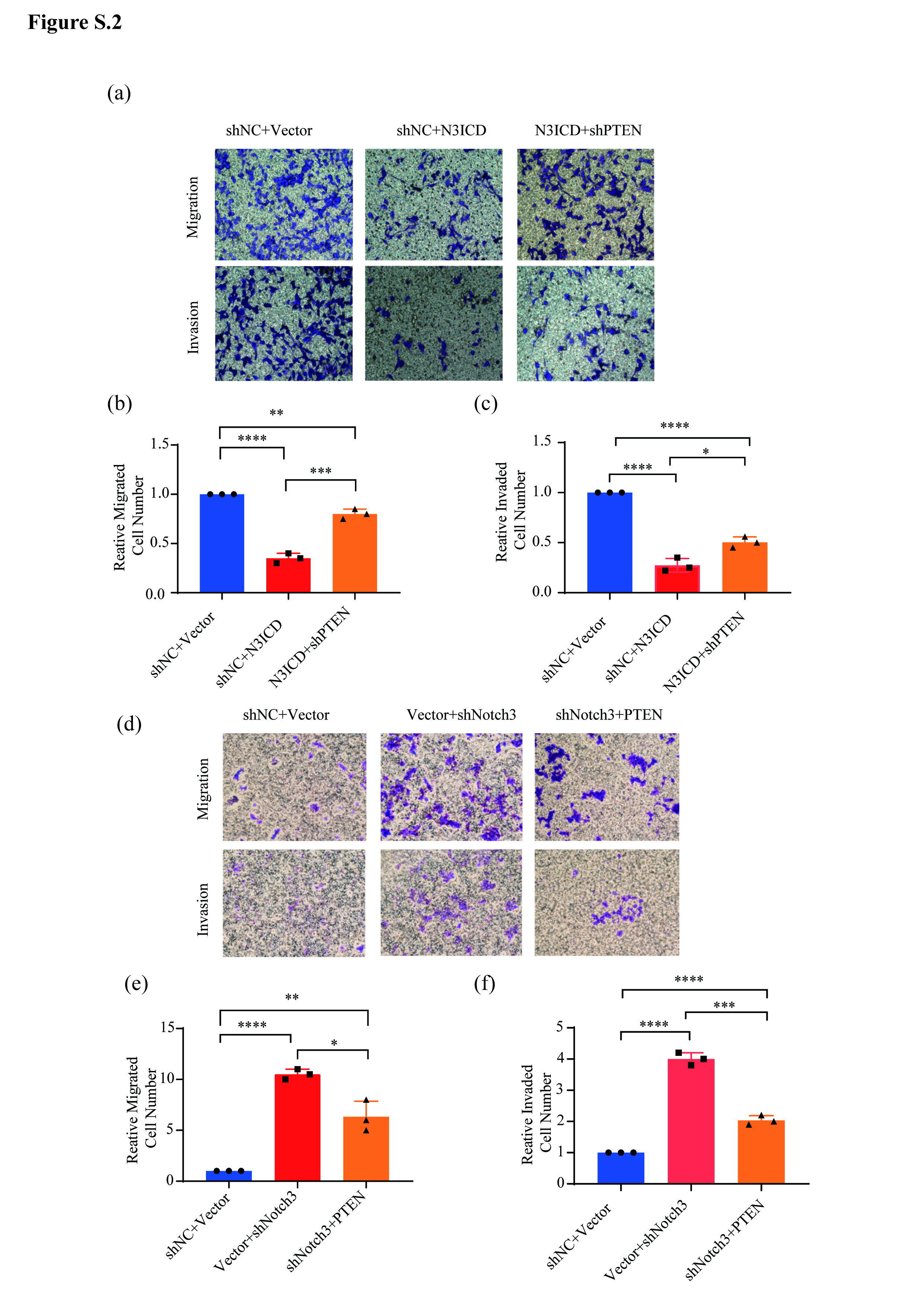

Supplement: Supplementary file 3 — F-Fig.S2 [file 41419_2021_3735_MOESM3_ESM.tif]

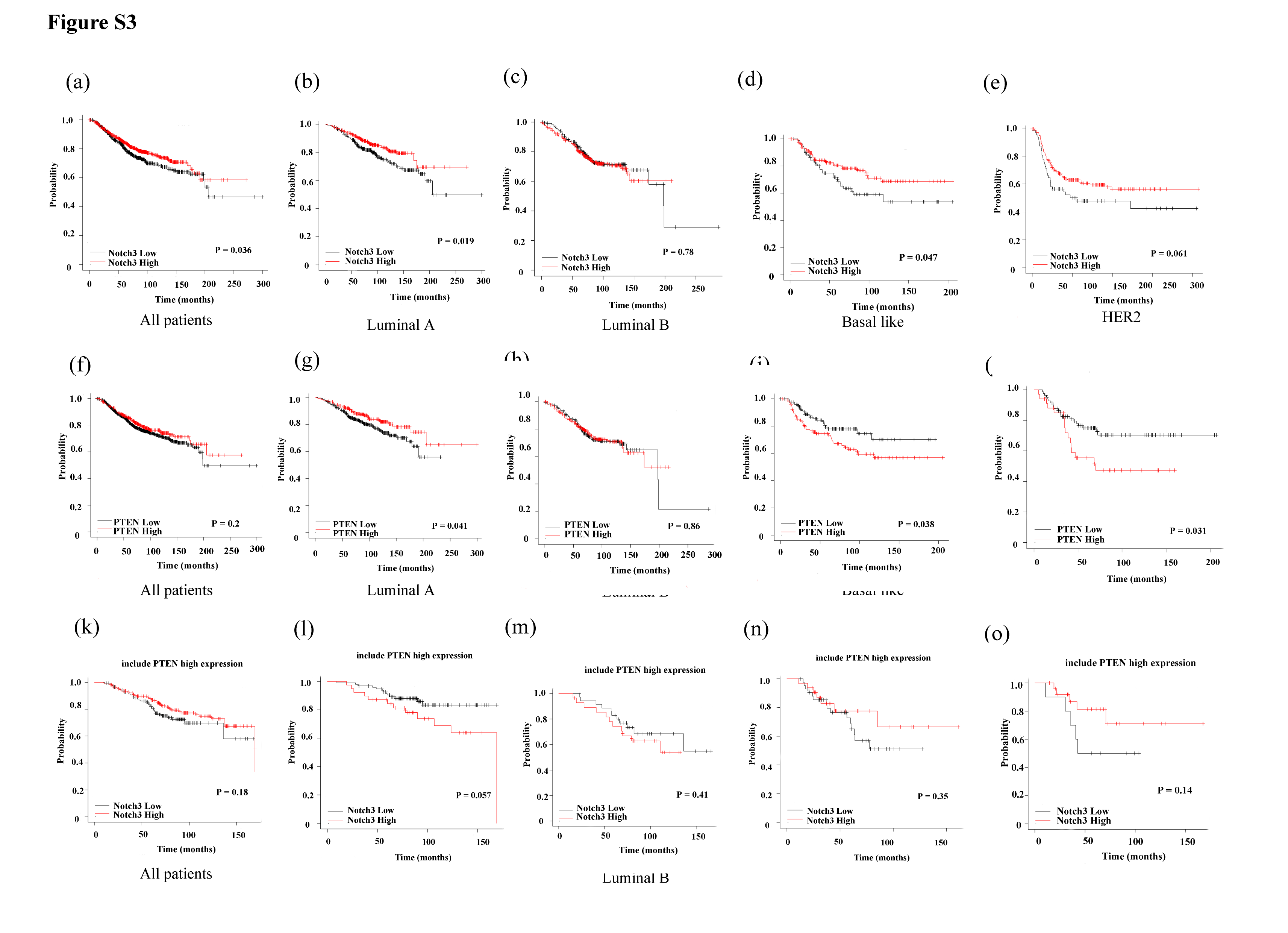

Supplement: Supplementary file 4 — F-Fig.S3 [file 41419_2021_3735_MOESM4_ESM.tif]
