# Supplementary material for: Notch3 inhibits cell proliferation and tumorigenesis and predicts better prognosis in breast cancer through transactivating PTEN
Source: Cell Death Dis. 2021 May 18;12(6):502. doi: 10.1038/s41419-021-03735-3 (PMC8131382; doi:10.1038/s41419-021-03735-3)
Supplement: Supplementary file 5 — Supplementary figure legend [file 41419_2021_3735_MOESM5_ESM.docx]

**Figure S1** **PTEN was highly expressed in luminal and ER-positive breast cancer subtypes based on GOBO analysis.**

**(a)** GOBO database analysis indicated that PTEN was highest level expressed in 25 types of Luminal breast cancer cell lines compared with 12 types of Basal A and 14 types of Basal B in this cohort (*P* = 0.0186). **(b)** PTEN was highest expressed level in 482 cases of Luminal A breast cancer and the total number was 5228 in this cohort compared the other subtypes (*P* < 0.00001). **(c)** PTEN was highly expressed level in ER-positive (1225 cases) compare with ER-negative (395 cases) breast cancer subtypes (*P* = 0.0106). ANOVA was used for statistical analysis.

**Figure S2 Ectopic Notch3 expression inhibits migration and invasion *in vitro*, which is attenuated by PTEN silencing**

**(a)** Stable N3ICD expression inhibited MDA-MB-231-luc cell migration and invasion *in vitro*. This effect was attenuated by PTEN silencing with shRNA. **(b, c)** Representative pictures and quantitative data from the migration and invasion assays are presented. **(d)** Stable Notch3 knockdown induced MCF-7 cell migration and invasion *in vitro*, which was attenuated by PTEN overexpression. (**e, f)** Representative pictures and quantitative data from migration and invasion assays are presented. **P* < 0.05, ***P* < 0.01, ****P* < 0.001, *****P* < 0.0001.

**Figure S3 Relationship between OS and Notch3 and PTEN mRNA expression in breast cancer subtypes**

High expression of Notch3 **(a, b, d)** and PTEN (**g**) had a better OS among different subtypes compared to the Notch3-low or PTEN-low groups. High expression of PTEN had a worse OS of breast cancer patients compared to the PTEN-low groups **(i, j)**. **(c, e)** Notch3 levels did not affect the OS of patients with the luminal B or HER2-positive breast cancer subtypes. PTEN levels did not affect the OS of all patients **(f)** or those with the luminal B subtype **(h)**. **(k-o)** Notch3 expression levels did not affect the OS of all high PTEN-expressing patients or those with high PTEN-expressing in the different subtype of breast cancer.
